# Of waves and troughs

**DOI:** 10.3389/fpsyg.2014.00197

**Published:** 2014-03-06

**Authors:** Michael Noll-Hussong

**Affiliations:** Department of Psychosomatic Medicine and Psychotherapy, University of UlmUlm, Germany

**Keywords:** mental disorders, neuroimaging, psychosomatic, cognitive neuroscience, affective neuroscience, social neuroscience

In 1998, *Eric Kandel* wrote in his intriguing paper titled “*A new intellectual framework for psychiatry*” (Kandel, 1998) that “the unique domain which psychiatry occupies within academic medicine, the analysis of the interaction between social and biological determinants of behavior, can best be studied by *also* having a full understanding of the biological components of behavior.” Fifteen years later, much like surfers who continue a frustrated and longing pursuit for the next “big one” (Cowan et al., 2000; Kandel, 2006), we are, according to *Henrik Walter*, in the midst of the third wave of biological psychiatry (Walter, 2013). Because a wave is, in a physical sense, a disturbance that propagates through space and time while transferring energy, there are at least three reoccurring “thermodynamic sinks” that I would like to *also* emphasize with *Walter* to ultimately better understand the complexity of the human brain in action (Bassett, 2011).

First is the rediscovery of the coequal contributions of emotions and affects toward normal brain functioning (Damasio, 2003; Tsuchiya and Adolphs, 2007). After *Michael Gazzaniga* and *George Miller* “invented” “*cognitive neuroscience*” in the late 1980s (Zorumski and Rubin, 2011), the predominance of a cognition-centered view of “higher” (and perhaps as one facet: more noble?) brain functions was able to again delay necessary and not so new “insults” to our species and misdirect (in its top-down-view of brain functioning) the conceptualization and treatment of mental disorders (Cromwell and Panksepp, 2011; Almada et al., 2013). Neurobiology helps us recalibrate the human wishful thinking we had come to appreciate regarding the “higher” and “lower” of the “conditio humana” imprinted in our (neuro)physiology. As the world divides into facts, there is in fact no *such* hierarchy imprinted in our brains. Rather, the brain seems to favor “dynamic coalitions of networks of brain areas with a high degree of *connectivity*,” and these networks - or the *connectome* - should not be conceptualized as being specifically affective or cognitive (Pessoa, 2008).

Second is the rediscovery of the body in biological psychiatry. *Walter* mentions the “4Es” (*embodiment, embeddedness, enactivism, extended cognition*) and the challenge of so-called “*situatedness*” (Walter, 2013). However, the very first step toward valuing the operant inter-wovenness of mind and body might be a simpler one. Interestingly of *ecto*dermal origin, neural tissue emerged enabling motor control in an evolutionary beneficial way. The brain originates in relation to a body that again, in relation to the outer world, actively moves – and, not least, gained the ability to interact with other bodies. Sensory information about the “*situation*,” the re-*flective* information involved in reflexes, is primarily able to close the loop and help coordinate movement. If *Antonio R. Damasio* is right, there is a need for *emotions* before we can *feel* anything, and these *emotions* are intimately connected with “more or less the complex reactions the *body* has to certain stimuli” (Damsio, 2005). These so-called “*somatic markers*” (Damasio, 1996) apparently make us capable of making predominantly beneficial decisions for self-preservation and the (we have to admit: biologically sexual) preservation of our species. It is *design*ative that the brain is the “*unmoved mover*.” However, changes are also reflected in the brain itself if the “motor-sensory” connections to the body are disturbed, e.g., in paraplegia (Wiens, 2005; Lenggenhager et al., 2012). The fantasy of an ever-dreaming, monolithic (but nonetheless self-conscious) “*brain in a vat*” that could reasonably think (or meaningfully simulate) about “*what is it like to be a bat*” (Nagel, 1974) currently suffers from not only solipsistic but also neurobiological-Darwinistic (so to say “inborn”) pitfalls. In this manifold context, it is interesting that today's “modern or third wave” of psychiatry is more willing to pay increased attention to enigmatic *somatic symptom disorders* (other than at first glance mere “brain disorders” such as *schizophrenia*, *depression*, *addiction*, and *dementia*) and attempts to incorporate the body and its imprinted neural representation into a genuine, more holistic understanding of the field. One could interpret it as a new esteem of anciently quirky psychosomatics in biological psychiatry that overcomes its centro-centric monodimensionality.

Third is the rediscovery of the importance of “being in relation” for reasonable neural functioning, especially in terms of social relationships for the human brain. From birth until death, human mammals need the “significant other(s),” and it is perhaps the most integrating framework covering cognitive and affective neurosciences that will give rise to emerging social neurosciences (Eisenberger and Cole, 2012; Singer, 2012). Newly emerging imaging techniques, such as *hyperscanning* (Babiloni and Astolfi, 2012), i.e., the simultaneous recording of brain activity of different subjects that allows “the study of inter-brain correlations between the cerebral activity of a group of interacting subjects as a unique system” (Babiloni and Astolfi, 2012), will help us understand the brain and perhaps pave the way to a central *second-person neuroscience* (Schilbach et al., 2013). Against this background, and only as one important example, empathy and the question of its quality and quantity in men have gained more and more attention in modern neuroscience (Gonzalez-Liencres et al., 2013). Psychotherapy and its proven impact on mental health (Etkin et al., 2005), before any technical question, fundamentally relies on the quality of the relation between two human beings (like patient and therapist) (Ardito and Rabellino, 2011). One could, again, interpret this rediscovery as the new esteem of anciently subordinate psychotherapy in biological psychiatry.

Finally, after three waves, a fourth wave seems inevitable. I would venture to predict that this “new wave” will belong to the computational neurosciences (Wen et al., 2011; Poldrack et al., 2012) and arise from the background of *information integration theory* (Tononi, 2005). The *Human Brain Project* (Markram, 2012) which was awarded one of the European Union's Flagship grants in 2013, worth more than €1 billion ($1.35 billion) over the next ten years, aims for the first time to *tie* or *link up* all knowledge of and to *simulate* the complete human brain from the molecule to the cortex on supercomputers to better understand how it functions (or even malfunctions), is ultimately the first step into a new era of real *cooperativeness* among neuroscientists and brains (Markram, 2013). Unfortunately, largely without “third wave” psychiatry. Just as affect and cognition, body and soul, the body-bound brain and *the* brain of my conspecific, and psychiatry and psychosomatics grow together, entities that belong together grow together. We have the opportunity to see the emergence of a new, non-reductionist science of fractal brains, as we examine mental orders and disorders differently, in a “brainy way,” with more cooperation and integration than ever before. In one word, in accordance with *Henry Markram* (Kandel et al., 2013): exciting!
